# The Association Between Added Sugars Intake and the Healthy Eating Food Index 2019 Among Canadians: Analyses from the 2015 Canadian Community Health Survey—Nutrition Public Use Microdata File

**DOI:** 10.3390/nu18152570

**Published:** 2026-08-06

**Authors:** Ye (Flora) Wang, Zhongqi Fan, Sandra Marsden, Chiara DiAngelo, Laura Chiavaroli, Mavra Ahmed, Mei Chung

**Affiliations:** 1 Nutrition Information Centre, Canadian Sugar Institute, Toronto, ON M5V 3E4, Canada; elienafan@hotmail.com (Z.F.); smarsden@sugar.ca (S.M.); cdiangelo@sugar.ca (C.D.); 2 Faculty of Land and Food Systems, University of British Columbia, Vancouver, BC V6T 1Z3, Canada; 3 Department of Nutritional Sciences, Temerty Faculty of Medicine, University of Toronto, Toronto, ON M5T 1R8, Canada; laura.chiavaroli@utoronto.ca (L.C.); mavz.ahmed@utoronto.ca (M.A.); 4 Toronto 3D Knowledge Synthesis and Clinical Trials Unit, Clinical Nutrition and Risk Factor Modification Centre, St. Michael’s Hospital, Toronto, ON M5B 1W8, Canada; 5 Li Ka Shing Knowledge Institute, St. Michael’s Hospital, Toronto, ON M5B 1W8, Canada; 6 Joannah and Brian Lawson Centre for Child Nutrition, University of Toronto, Toronto, ON M5T 1R8, Canada; 7 Friedman School of Nutrition Science and Policy, Tufts University, Boston, MA 02111, USA; mei_chun.chung@tufts.edu; 8 Nutrition Evidence Synthesis and Data Analysis LLC, Boston, MA 02135, USA

**Keywords:** added sugars, free sugars, diet quality, healthy eating food index, beverage, food sources

## Abstract

Background/Objectives: There is continuing debate regarding the association between added sugars (AS) consumption and overall diet quality, which remains a research gap within the Canadian population. This study aimed to assess the association between intakes of added sugars and the Healthy Eating Food Index (HEFI) 2019 that measures adherence to Canada’s Food Guide as an indicator of diet quality. Methods: The first 24 h dietary recalls of Canadians 2 years and older (*n* = 19,532) from the 2015 Canadian Community Health Survey—Nutrition were used. Added sugars intakes were calculated based on a systematic 10-step algorithm. Descriptive statistics were obtained for total HEFI and component scores across different AS intake categories. The association between AS categories and HEFI scores was tested using a generalized linear model adjusted for age, sex, education level, type of smoker, and misreporting status. Results: The association between continuous AS intake and HEFI scores was non-linear. In the categorical model, AS intake categories were inversely associated with HEFI scores among Canadians (R^2^ = 0.32, *p* < 0.001). Large overlaps in HEFI scores were observed among individuals with similar AS intakes for the whole population and each age group. Among high AS consumers (>15% EI), lower HEFI scores were largely due to higher proportions of sweetened beverages (i.e., low beverage scores) and lower intakes of vegetables and fruit and plant-based proteins. Scores for protein food and whole grains were low across all AS intake categories. Conclusions: Large variations in overall HEFI scores were observed amongst those with similar AS intakes, suggesting that added sugars alone may not fully capture overall diet quality or adherence to Canada’s Food Guide.

## 1. Introduction

In recent years, the global food industry has been under increasing pressure to reduce the sugar content in products, driven by concerns about the health impacts of excessive sugar consumption. Excessive intakes of added or free sugars, particularly sugar-sweetened beverages, have been associated with a range of chronic conditions, including obesity, type 2 diabetes, cardiovascular diseases, and dental issues [1,2,3]. In 2015, the World Health Organization free sugars guideline recommended limiting the consumption of free sugars to less than 10% energy intake (EI), based on low to moderate observational evidence linking free sugars with dental caries [4,5]. This was followed by other government bodies and health organizations adopting similar guidelines [6,7].

In Canada, the 2019 Canada’s Food Guide (CFG) provides Canadians with comprehensive guidance on dietary choices and eating patterns [8]. Although the CFG does not set a specific limit on added sugars or free sugars, the dietary guidance recommends choosing foods with little to no added sugars and choosing water instead of sugar-sweetened beverages. The Healthy Eating Food Index (HEFI) was established to assess the extent to which Canadians’ food choices are aligned with the 2019 CFG recommendations (“what to eat”) [9]. Higher HEFI scores correspond to better alignment with healthy food choice recommendations, and overall higher diet quality. The free sugars component score within the HEFI algorithm uses scoring thresholds that align with the WHO recommendation. A maximum free sugars score of 10 (out of a total of 80) is defined as free sugars intake <10% EI. It is worthwhile to note that even though the WHO 10% EI free sugars threshold that HEFI adopted was based on its association with dental caries rather than diet quality, HEFI is the only dietary index established to assess Canadians’ eating patterns in compliance with the Food Guide. On the contrary, the Dietary Guidelines for Americans 2020–2025 had sugar-related recommendations grounded in nutrient adequacy. It recommends limiting added sugars to below 10% EI, based on food pattern modeling that identifies the number of discretionary calories available after meeting essential nutrient needs [1]. Interestingly, research based on the U.S. NHANES dietary survey suggests that intakes below approximately 18% EI from added sugars were generally not associated with micronutrient inadequacy among U.S. adults [10]. While a recent systematic review reported that higher intakes of added or free sugars were generally associated with lower diet quality, it also suggested lower to moderate intakes of sugars may have different magnitudes or directions of association compared to higher intakes of sugars [11]. Challenges also remain in differentiating the impacts of added sugars from various food and beverage sources, taking into account their nutrient profile and the broader dietary pattern in which they are consumed.

As a result, this study aimed to explore the association between added sugars intake and HEFI scores as a marker for diet quality among Canadian adults and children. Considering the context in which sugars are consumed and the broader dietary patterns they reflect, it was hypothesized that substantial variation in HEFI-2019 scores would be observed among individuals with similar added sugars intakes.

## 2. Materials and Methods

### 2.1. Data Source

The 2015 Canadian Community Health Survey (CCHS)—Nutrition is a nationwide, comprehensive survey on nutrition, conducted from January to December 2015 [12]. Even though the data is over 10 years old, and current dietary patterns may have changed considerably from those observed at that time, it remains the most current dietary survey data available in Canada. Briefly, nutrition-related information was collected using a 24 h dietary recall, administered in person in 10 Canadian provinces by skilled interviewers using a computerized version of the Automated Multiple-Pass Method from the U.S. Department of Agriculture. The target population for the 2015 CCHS—Nutrition includes all individuals aged 1 year and above living in private dwellings in the 10 Canadian provinces, and did not include individuals who were full-time members of the Canadian Forces or who lived in the Territories, on reserves and other Aboriginal settlements, in some remote areas, or in institutions (e.g., prisons or care facilities). After the initial 24 h recall (*n* = 20,487), a random subsample of 7608 participants completed a second recall via phone 3–10 days later. Additionally, data on socio-demographic attributes, body measurements, and health conditions were collected through a detailed, interviewer-led health questionnaire. Interviews were proxy-reported for children aged 1–5 years, parent-assisted for those aged 6–11 years, and self-reported for respondents aged ≥12 years [12].

**Figure 1:**
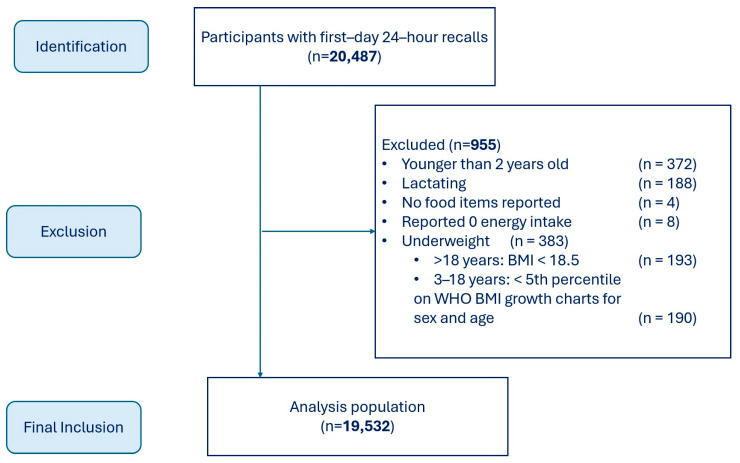
Study population description flow chart.

The study’s sampling methodology, designed to reflect the Canadian population by considering factors such as age, gender, location, and socioeconomic background, is described elsewhere [12]. Data were excluded from the analysis if participants were younger than 2 years old, were lactating, had no food items reported or reported 0 energy intake, or were underweight (Figure 1), defined as a BMI < 18.5 for individuals older than 18 years and less than the 5th percentile on the World Health Organization (WHO) BMI growth charts for sex and age for individuals between 3 and 18 years old. Underweight individuals (approximately 2.0% of the analytic sample) were excluded from the analysis because validated IOM equations for estimating energy expenditure are not available for this population [13], and estimating energy expenditure is required to assess the plausibility of participants’ reported energy intake (i.e., energy misreporting). The 2015 CCHS—Nutrition Public Use Microdata File (PUMF) does not identify pregnant participants; therefore, the pregnant population could not be excluded from the analysis. After exclusions, the analysis population consisted of 19,532 participants. Foods and beverages reported in the 24 h dietary recall were coded by Health Canada using the 2015 Canadian Nutrient File and Health Canada Bureau of Nutritional Science (BNS) food codes [12].

### 2.2. Estimation of Added Sugars and Free Sugars

To estimate the intakes of added sugars and free sugars, a 10-step algorithm was applied [14]. In this process, the term “added sugars” is defined as sugars and syrups that are added to food during its production or consumption. For the definition of “free sugars”, the WHO definition was used, which includes sugars added to foods by manufacturers or consumers, as well as those naturally present in honey, syrups, and fruit juices. In practical terms, free sugars are equivalent to the combination of added sugars and sugars present in 100% fruit juices. For each individual BNS food code (*n* = 177, Supplementary Table S1), added and free sugars were estimated according to the 10-step algorithm (Supplementary Table S2). The majority of estimations (94%) were made objectively, as defined in steps 1–6; approximately 6% were determined more subjectively, using other available data, assumptions, and estimates as described in steps 7–10. Sugars present in fruits, vegetables, and unsweetened milks were considered naturally occurring. All sugars in confectionery, sugars and syrups, and baked goods and products were categorized as added sugars. For foods such as sweetened yogurts, the amount of added sugars was determined by calculating the difference between the total sugar amount in the sweetened version and its unsweetened counterpart. For fruit drinks and frozen desserts, even though they contain both naturally-occurring and added sugars, the proportion of naturally-occurring sugar-containing ingredients cannot be accurately determined based on the information available in CCHS; therefore, added sugars were assigned based on the total sugar amount even though it is an overestimation. Consumption of added sugars (AS) was calculated as grams per day (g/day) and a percentage of total energy intake (EI) per day (AS %EI) using the formula (AS in grams × 4 kcal/g) / total EI. The study population was then divided into categories based on AS intake thresholds: less than 5% EI, 5–10% EI, 10–15% EI, 15–20% EI, and above 20% EI. The cut-off values between categories took into consideration the DGA 10% EI recommendation for added sugars, and the 5% EI increments were determined to allow for adequate differentiation across the entire range of intake levels. Age categories were consistent with Health Canada’s Dietary Reference Intake (DRI) age groups: 2–8 years, 9–13 years, 14–18 years, 19–30 years, 31–50 years, and over 50 years [15].

### 2.3. Energy Misreporting

Due to the self-reporting nature of the data, the CCHS—Nutrition is subject to misreporting, including both underreporting and overreporting of dietary intake. Based on the method proposed by Garriguet [13], each respondent’s reported EI was compared to their total energy expenditure (TEE) to identify misreporters. Underreporting and overreporting were defined as having an EI:TEE ratio of <0.7 and >1.42, respectively; the ratios in-between were considered plausible reporting. A fixed physical activity level was assumed across the population, categorizing respondents younger than 14 years old as low active, and those 14 years and older as sedentary. For respondents with valid measurements of weight and height, their TEE was calculated using equations from the Institute of Medicine [15]. These equations take into account each individual’s age, sex, and BMI. BMI was determined by dividing measured weight (in kilograms) by the square of measured height (in meters). Adults aged 18 and older were classified as underweight if their BMI was below 18.5 kg/m^2^, normal weight with a BMI ranging from 18.5 to 24.9 kg/m^2^, and overweight or obese with a BMI above 25 kg/m^2^ [16]. For those younger than 18, the Centers for Disease Control and Prevention growth charts were used, with below the 5th percentile and above the 95th percentile as cut-off points for underweight and overweight, respectively [15,17]. Since the IOM equations are not applicable to individuals classified as underweight, a sensitivity analysis was conducted to show that excluding the underweight population (accounting for 2% of the sample) resulted in the same level of significance and similar beta-coefficients in the association between added sugars categories and HEFI-2019 scores (Supplementary Figure S1). As a result, the underweight individuals were excluded from the main analyses so as to account for energy misreporting. For respondents lacking valid height and weight measurements, their TEE was estimated based on age, sex, and activity level, employing the “Energy levels for assignment of individuals to USDA Food Patterns” standards [18,19].

### 2.4. The Healthy Eating Food Index 2019 (HEFI-2019)

Canada’s Food Guide (CFG)-2019 provides dietary guidance to assist Canadians in meeting their nutrition needs, improving their health, and reducing the risk of nutrition-related chronic diseases [8]. The HEFI-2019 was developed using data from the 2015 CCHS—Nutrition to assess the extent to which individuals’ eating patterns align with the healthy food choices outlined in CFG-2019. The process and methods for constructing the HEFI-2019 are described elsewhere [9]. Briefly, the index comprises 10 food and nutrient-based components with a total point score of 80, where higher points indicate greater alignment with CFG-2019 (Supplementary Table S3). The 10 components include vegetables and fruits, whole-grain foods, grain foods ratio, protein foods, plant-based protein foods, beverages, fatty acids ratio, saturated fats, free sugars, and sodium. Each component corresponds to key recommendations outlined in CFG-2019.

### 2.5. Statistical Analyses

All analyses were conducted using SAS version 9.4 (SAS Institute, Cary, NC, USA). Selected figures were generated using R version 4.3.3 (R Foundation for Statistical Computing, Vienna, Austria). To ensure the results are representative on a nationwide scale, survey weights provided by Statistics Canada were applied as appropriate. All measures of variance were estimated using the bootstrap balanced repeated replication method, with 500 replicate weights, to account for the complex survey design of the 2015 CCHS. Weighted added sugars %EI categories were reported and compared across different age and HEFI groups. In addition, three different energy adjustment methods were tested in the analysis: the nutrient density model, the residual model, and the energy partition model (Supplementary Table S4). The nutrient density model was chosen as the best approach for energy adjustment based on R^2^, AIC and BIC values.

Our analysis involved analyzing added sugars as continuous (AS %EI) or categorical variables to explain the nature of the relationship between added sugars intake and the HEFI score (Supplementary Table S5). Specifically, to evaluate the relationship between added sugars consumption and HEFI scores, a general linear model (GLM) was fitted with HEFI score and added sugars intake adjusted for age, sex, education, misreporting status, and type of smoker. The results and residual plots were used to determine the linear/non-linear relationship between dependent and independent variables (Supplementary Figure S2). A quadratic term for AS %EI was also added to further evaluate potential non-linearity in the relationship. A statistically significant quadratic term was interpreted as evidence that the association between AS %EI and HEFI-2019 scores was not adequately described by a simple linear relationship. The goodness-of-fit for each model was evaluated by comparing the coefficient of determination (R^2^), Akaike Information Criterion (AIC), and Bayesian Information Criterion (BIC) values (Supplementary Table S6). In the end, the GLM model that had the best fit based on model fitness statistics was selected, i.e., the model evaluating the relationship between added sugars %EI categories and HEFI-2019 scores, adjusted for age, sex, education, misreporting status, and type of smoker. In the final model, the bootstrap balanced repeated replication method with 500 replicate weights was applied to make the results represent the whole population. A *p*-value of less than 0.05 was considered statistically significant.

Since the percentage of energy from added sugars (AS %EI) was skewed to the left, with a maximum of 100 for AS %EI, a sensitivity analysis was conducted to evaluate the effect of outliers on the study results. A model was fitted excluding participants who had added sugars over 40% EI (*n* = 151, 0.77% of the study population), and the results were comparable with those from the final model (Supplementary Figure S3).

## 3. Results

### 3.1. Sample Characteristics

The survey population analyzed in this study consisted of 19,532 individuals (51.9% females), ranging from 2 to 95 years old, with an average age of 39.8 years. The key demographic information of the study sample and their associations with HEFI are described in Supplementary Tables S7–S9.

### 3.2. Added Sugars Consumption

**Figure 2:**
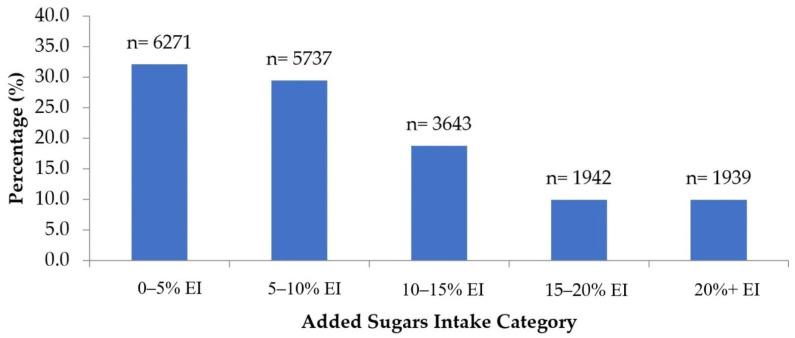
Weighted distribution of the population by added sugars intake categories in 5% EI increments (*n* = 19,532).

The weighted mean added sugars intake was 9.7% EI, with higher mean intakes among adolescents and lower mean intakes among older adults (Table 1). Females had higher added sugars intakes as %EI than males in all age groups except 2–8 y and 31–50 y. The populations were also categorized into five groups based on added sugars intakes (% EI) in 5% EI increments (Figure 2). About 65% of the study population had added sugars intakes less than 10% of their energy intake, a level recommended by the Dietary Guidelines for Americans 2020–2025.

### 3.3. HEFI-2019 by Added Sugars Intakes

**Figure 3:**
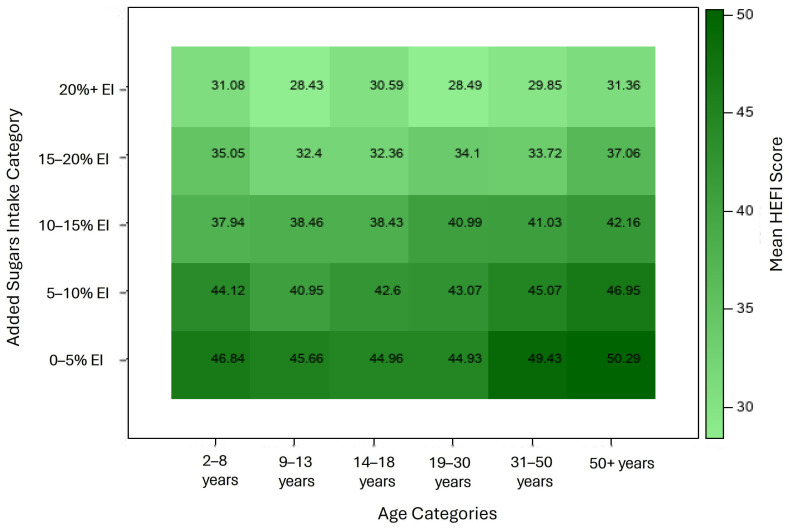
Heat map of weighted mean HEFI scores by sub-age groups and AS intake categories.

The mean HEFI-2019 score in the study population was 42.4 ± 11.6 (Standard Deviation), ranging from 2.4 to 77.6. Within each added sugars category, a general trend was observed that the mean HEFI score was lower among adolescents and higher among older adults (Figure 3). Compared to individuals with 0–5% EI from added sugars, individuals with 5–10% EI, 10–15% EI, 15–20% EI, and 20+% EI had a mean decrease in HEFI-2019 score of 3.22, 7.43, 13.18, and 17.52, respectively (Table 2), suggesting a negative association (r^2^ = 0.32, *p* < 0.001) between added sugars intake and total HEFI-2019 scores after adjusting for age, sex, education level, type of smoker, and misreporting status. The associations were attenuated when analyses were performed between added sugars intake and HEFI-2019 scores without the free sugars component (r^2^ = 0.14, *p* < 0.001), and HEFI-2019 scores without both free sugars and beverage components (r^2^ = 0.10, *p* < 0.001, Table 2).

**Figure 4:**
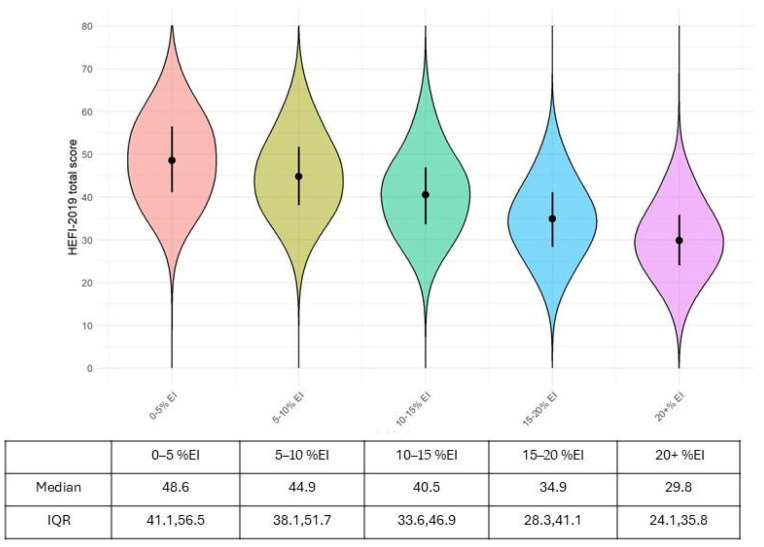
Weighted distribution of HEFI scores (with medians and interquartile ranges) across AS intake categories among Canadians aged 2 years and older.

The weighted distributions of HEFI scores across AS intake categories are shown in Figure 4 for different age subgroups. Within each AS intake category, there was a wide distribution of HEFI scores, with significant overlaps across different AS intake categories. Similar trends were observed among sub-age groups (Supplementary Figure S4).

### 3.4. Added Sugars and HEFI Component Scores

**Figure 5:**
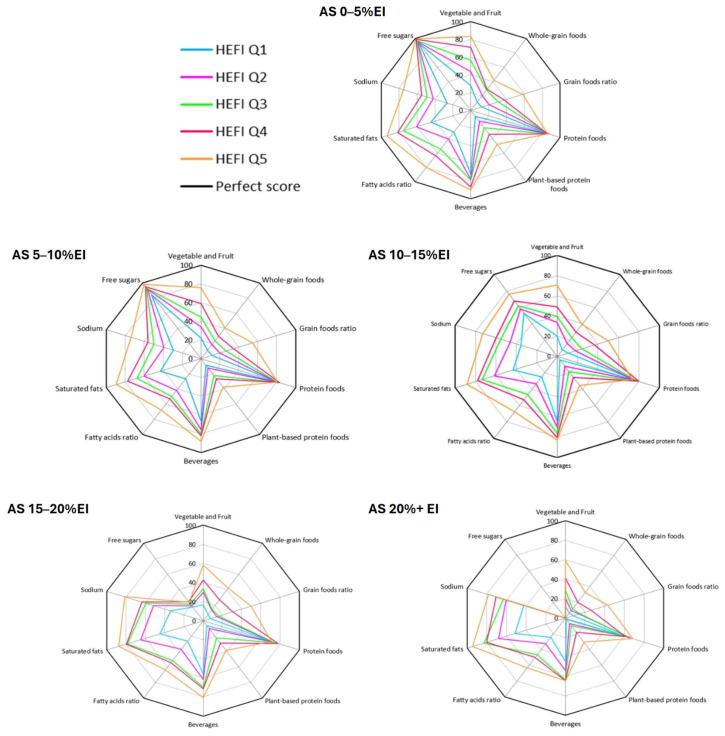
Spider plots showing HEFI component scores (% to the max score) within each added sugars intake category.

While the free sugars HEFI scores were similar within each AS intake category, greater differences were observed in the scores for fruits and vegetables, sodium, saturated fat, fatty acid ratio, and grain foods ratio (Table 3, Figure 5). Interestingly, there were minimal variations in protein food intakes, suggesting a stable contribution across both HEFI quintiles within each category and across added sugars intake categories. When comparing HEFI scores across added sugars intake categories, the beverage scores remained stable and relatively high (>60% of max score) in the three intake categories (0–5% EI, 5–10% EI and 10–15% EI), suggesting the variation in added sugars intakes were largely from various food sources of added sugars rather than beverages. Starting from the 15–20% EI category, the patterns clustered toward the centre (i.e., lower scores) for several components such as beverages, plant-based protein foods, and vegetable and fruit scores. The whole-grain foods, grain foods ratio, and plant-based protein foods scores remained generally low across all AS intake categories (Table 3), even for those in the highest HEFI quintile (Figure 5).

## 4. Discussion

Most global and regional dietary guidelines on reducing intakes of added or free sugars have been based on research literature in relation to obesity, dental caries or other disease outcomes. For example, the World Health Organization (WHO) 10% free sugars guideline was based on observational research assessing the association between free sugars intake and dental caries incidence [4]. The European Food Safety Authority (EFSA) recommends limiting the intakes of free sugars as low as possible within a nutritionally adequate diet based on major chronic disease risk factors [6]. However, EFSA used relative free sugar exposure calculations, making it difficult to draw conclusions on real-life consumption levels that are compatible with this recommendation [6,19]. An exception is the Scientific Report of the Dietary Guidelines for Americans 2020–2025, which determined by menu-modeling that when added sugars intake is over 10% of energy, nutrient adequacy may not be achieved without over-consuming calories [1]. In the Canadian context, our study is the first to investigate the association between intakes of added sugars and diet quality, using HEFI-2019 scores as an indicator of adherence to the 2019 Canada’s Food Guide across different age groups in both children and adults. Results suggest that the association was negative and non-linear, with the greatest decline in HEFI-2019 scores at the highest level of added sugars intakes (20+ %EI).

This observation is similar to a recent systematic review and meta-analysis, which reported negative associations between added sugars and diet quality indexes such as the U.S. adjusted Healthy Eating Index-2010 and Global Diet Quality Score [11]. In the present study, assessment of the continuous added sugars exposure indicated that its association with diet quality was non-linear. Based on this finding, added sugars intake was subsequently modeled as a categorical variable to better characterize differences in diet quality across intake levels. The non-linear pattern observed in this study may provide additional insight, as many of the studies included in the previous systematic review and meta-analysis did not specifically assess potential non-linear relationships. As shown in the spider plot of HEFI component scores (Figure 5), the magnitudes of contributions of different food/nutrient components to total HEFI scores remained fairly similar among those with low to moderate intakes of added sugars (<15% EI). In contrast, the patterns for the two highest added sugars intake groups (15–20% EI and 20+% EI) were clearly distinct. Lower HEFI scores for the top two AS intake groups were largely attributed to reduced scores in beverages, plant-based proteins and vegetable and fruits, highlighting key areas for dietary improvement in these individuals. Therefore, this research not only addresses a gap in the literature regarding the association between added sugars and adherence to Canada’s Food Guide in the Canadian population but also suggests high variability in the spectrum of eating patterns across different intakes of added sugars. This pattern may also be partially explained by the scoring criteria of the free sugars component in HEFI, with free sugars intakes below 10% EI getting the maximum score of 10. Given the fact that added sugars as the independent variable is partially connected to free sugars and sugar-sweetened beverages in the HEFI scoring algorithm, additional analyses were performed to dissect the connection by excluding free sugars and beverage component scores from the total HEFI score. Indeed, the beta-coefficients were attenuated between added sugars and HEFI residual scores (excluding the free sugars component, and excluding both free sugars and beverage components), suggesting that the reported association between added sugars and HEFI is partially inflated by this proximity in algorithm. Previous data from the U.S. National Health and Nutrition Examination Survey (NHANES) 2009–2014 also reported non-linearity but with a threshold of 18% EI, below which added sugars were not associated with nutrient inadequacy [10]. Collectively, the unique non-linear pattern observed between added sugars and diet quality markers underscores the need for future investigations into non-linear regression analyses on the association between sugars and diet quality (especially when using diet quality indexes) to capture potential complexities and nuances that linear frameworks may overlook. Such an approach is similar to the proposed future consideration in the umbrella review on added sugars, sugar-sweetened beverages and chronic disease outcomes as part of the Scientific Foundation for the Dietary Guidelines for Americans 2025–2030 [20].

In this study, large variations in HEFI scores were also observed among Canadians with similar intakes of added sugars, suggesting that added sugars alone may not fully capture overall diet quality or adherence to Canada’s Food Guide. Reducing added sugars for better health outcomes is more nuanced than an oversimplified “less is better” approach. Analysis of the NHANES 2011–2018 shows that with increasing AS calories, the contribution of “soft drinks,” “fruit drinks,” and “tea” to AS increased, whereas the contribution of ready-to-eat cereals to AS decreased in both children and adults [21]. While higher added sugars intakes from soft drinks, fruit drinks and coffee and tea were associated with lower HEI scores and most of the HEI components, higher added sugars intakes from flavoured milk and snack/meal bars among children, and from sweet bakery products among adults, were associated with higher HEI scores [22]. Collectively, food-source based research findings emphasize the complexity of food choices that influence diet quality rather than focusing on a single nutrient.

Another key observation in this study is that lower HEFI scores observed for the 15–20% EI and 20+% EI categories were partially attributed to lower scores in beverages (representing higher proportions of sweetened beverages in total beverage consumption) as well as lower scores for plant-based proteins and vegetable and fruits. This pattern supports a food-specific dietary pattern in line with Canada’s Food Guide, with a focus on encouraging the consumption of unsweetened beverages. This approach is echoed by global efforts through front-of-package labelling regulations, as well as government-led or industry-voluntary sugar reduction strategies to encourage reduced consumption of sugar-sweetened beverages. Over the past decades, Canadian consumption of sugar-sweetened beverages has been the major driver for declining consumption of added sugars [23], a trend also observed in the United States and many other countries [24,25].

This is the first study that utilized the Canadian national dietary survey to assess the association between added sugars intakes and diet quality using a newly developed index, HEFI-2019, which measures the alignment of individual eating patterns with Canada’s Food Guide 2019. Additional analysis of the component score variations provides insights towards different food choices for individuals at different levels of added sugars consumption to help inform future government policy development as well as personalized dietary guidance by registered dietitians. This study has several limitations. Firstly, the intakes of added/free sugars in beverages are penalized in two HEFI components (i.e., free sugars score and beverage score), which further exacerbates the circularity between the independent and dependent variables. Secondly, there is currently no standardized, up-to-date Canadian nutrient database for the added sugars content of foods and beverages. As a result, added sugars intake was estimated using established methodology that incorporates a proportion of subjective calculation, introducing additional uncertainty into the accuracy of these estimates. Further, the CCHS-2015 and Canadian Nutrient File dataset, even though the most recent nationwide dietary survey available, is now more than a decade old. Current dietary patterns may have changed considerably from those observed at that time, influenced by shifts in consumer behaviour during and after the COVID-19 pandemic and increasing sugar reduction/reformulation efforts in the marketplace in response to government policies (such as front-of-package labelling regulations). The use of a single 24 h dietary recall rather than usual intake data further limits the accuracy of dietary intake estimates. A single 24 h recall provides only a snapshot of one day’s intake, so it cannot capture day-to-day variability or habitual eating patterns. It also relies heavily on memory and self-report, making it prone to recall errors, underreporting, and portion-size inaccuracies, which introduce measurement bias. However, the 24 h recall is less prone to social desirability bias compared to other dietary recall methods. We have also attempted to account for misreporting status in our analysis. HEFI-2019 was used as a surrogate marker for diet quality, which has its own limitations, as described elsewhere [9]. It is designed to reflect guidance for the generally healthy population but does not capture specific dietary guidance for population subgroups, such as pregnant women, individuals with specific dietary requirements or individuals requiring specialized diets in a clinical setting. In this study, we assumed that higher alignment with Canada’s Food Guide 2019 is associated with higher diet quality, as it has a strong correlation with the Healthy Eating Index 2015. However, HEFI-2019 so far has been validated against nutrient intakes only in certain populations [26]. Finally, even with adjustments for variables available in the dataset, analyses of the relationship between intake of added sugars and HEFI are complicated by measurement error, unmeasured or imperfectly measured residual confounding (such as cultural food practices, socioeconomic constraints, food access, cooking skills, and health-related behaviours) and other biases inherent in dietary assessment tools, as well as the approaches used to account for the influence of total energy intake on the associations examined.

## 5. Conclusions

While a negative association was observed between added sugars intakes and HEFI scores, substantial variations in HEFI-2019 scores within AS intake categories suggest that added sugars intake alone may not fully capture overall diet quality or adherence to Canada’s Food Guide as assessed by HEFI-2019. This research highlights the need to consider multi-dimensional food choices in the context of diet quality.

## Figures and Tables

**Table 1:** Weighted added sugars (AS) intakes in grams and %EI by age and sex group.

Age Group	Both		Male		Female	
	*n*	Mean (95% CI)	*n*	Mean (95% CI)	*n*	Mean (95% CI)
AS %E						
2–8 y	2126	9.4 (8.8, 9.9)	1075	9.69 (9.0, 10.4)	1051	9.08 (8.3, 9.8)
9–13 y	1942	11.0 (10.4, 11.7)	1012	10.93 (10.2, 11.6)	930	11.16 (10.2, 12.1)
14–18 y	1917	10.9 (10.0, 11.8)	919	10.50 (9.2, 11.8)	998	11.21 (10.3, 12.1)
19–30 y	1775	9.8 (8.7, 10.9)	859	9.38 (8.0, 10.8)	916	10.33 (9.2, 11.5)
31–50 y	4129	8.8 (8.4, 9.2)	1941	9.10 (8.5, 9.7)	2188	8.45 (7.8, 9.1)
50+ y	7643	8.4 (8.0, 8.8)	3587	8.16 (7.7, 8.7)	4056	8.54 (8.0, 9.1)
AS grams/day						
2–8 y	2126	38.1 (35.2, 40.9)	1075	40.6 (37.2, 44.1)	1051	35.7 (31.4, 40.0)
9–13 y	1942	54.9 (51.1, 58.7)	1012	57.9 (52.8, 63.1)	930	51.6 (46.8, 56.3)
14–18 y	1917	58.9 (54.0, 63.8)	919	65.9 (58.2, 73.7)	998	51.5 (46.1, 56.8)
19–30 y	1775	50.8 (42.4, 59.1)	859	58.1 (49.1, 67.1)	916	42.0 (32.6, 51.4)
31–50 y	4129	43.1 (40.6, 45.7)	1941	51.3 (47.5, 55.1)	2188	34.7 (31.5, 37.9)
50+ y	7643	37.7 (34.8, 40.6)	3587	42.2 (38.7, 45.6)	4056	33.6 (29.8, 37.5)

**Table 2:** Generalized Linear Model’s coefficient, 95% CI, and *p*-value between added sugars (AS) categories, HEFI, HEFI without free sugars, and HEFI without free sugars and beverage components.

		HEFI		HEFI Without Free Sugars		HEFI Without Free Sugars and Beverages	
		Estimate	95% CI	Estimate	95% CI	Estimate	95% CI
Intercept		44.31	43.36, 45.26	35.24	34.26, 36.22	27.95	27.28, 28.61
AS categories	0–5% EI (reference)	0	0	0	0	0	0
	5–10% EI	−3.22 *	−3.92, −2.51	−2.77	−3.45, −2.10	−2.63	−3.17, −2.07
	10–15% EI	−7.43 *	−8.16, −6.70	−4.14	−4.90, −3.38	−5.70	−6.92, −4.48
	15–20% EI	−13.18 *	−15.06, −11.31	−5.65	−7.46, −3.84	−4.44	−5.78, −3.11
	20+% EI	−17.52 *	−19.20, −15.85	−7.87	−9.54, −6.20	−3.58	−4.20, −2.97
		r^2^ = 0.32 *		r^2^ = 0.14 *		r^2^ = 0.10 *	

* *p* < 0.001.

**Table 3:** Mean HEFI component scores across added sugars intake categories.

Component	<5% EI	5–10% EI	10–15% EI	15–20% EI	20+% EI	Maximum Score
Vegetable and Fruit	10.98	9.24	8.33	7.36	5.99	20
Whole-grain foods	1.37	1.33	1.17	1.03	0.77	5
Grain foods ratio	1.61	1.51	1.35	1.21	0.99	5
Protein foods	4.31	4.11	3.79	3.63	3.14	5
Plant-based protein foods	1.11	1.02	0.91	0.8	0.66	5
Beverages	8.24	7.88	7.29	6.6	5.67	10
Fatty acids ratio	2.52	2.25	2.21	2.22	2.14	5
Saturated fats	3.38	3.29	3.4	3.49	3.76	5
Sodium	4.82	5.03	5.47	5.79	6.51	10
Free sugars	9.68	9.1	6.27	2.00	0	10

## Data Availability

The original data presented in this study are openly available from the Statistics Canada 2015 Canadian Community Health Survey (CCHS)—Nutrition Public Use Microdata File (PUMF) (product number:82M0024X2018001). The derivation and statistical analysis codes are available from the corresponding author upon reasonable request.

## Supplementary Materials

The following supporting information can be downloaded at https://www.mdpi.com/article/10.3390/nu18152570/s1, Sensitivity analysis with or without participants with added sugars greater than 40% EI; Table S1. Food categories by BNS codes. Table S2. The 10-step algorithm to estimate added sugars, adapted from Louie et al. [14]. Table S3. HEFI component score algorithm (adapted from Brassard et al. 2022 [9]); Table S4. Energy adjustment model selection; Table S5. The regression models tested for the relationship between added sugars and HEFI scores; Table S6. Regression model selection results; Table S7. Key demographic characteristics of the study population; Table S8. Distribution of the study population by added sugars intake categories; Table S9. Generalized Linear Model’s coefficient, %95 CI, and *p*-value between HEFI residual scores and added sugar (AS) categories, and key demographic groups in the regression model. Figure S1. Sensitivity analysis results with underweight population included. Figure S2. Non-linear relationship between HEFI and variables (% from added sugars and age). Figure S3. Sensitivity analysis with or without participants with added sugars greater than 40% EI. Figure S4. Weighted distribution of HEFI scores (with medians and interquartile ranges) across AS intake categories and age groups. References [9,14] are cited in the supplementary materials.

## Abbreviations

The following abbreviations are used in this manuscript:

AS	Added sugars
BMI	Body mass index
BNS	Bureau of Nutritional Science
CCHS	Canadian Community Health Survey
CFG	Canada’s Food Guide
EFSA	European Food Safety Authority
EI	Energy intake
HEFI	Healthy Eating Food Index
NHANES	National Health and Nutrition Examination Survey
PUMF	Public use microdata file
TEE	Total energy expenditure
WHO	World Health Organization
