# Supplementary material for: The Association Between Added Sugars Intake and the Healthy Eating Food Index 2019 Among Canadians: Analyses from the 2015 Canadian Community Health Survey—Nutrition Public Use Microdata File
Source: Nutrients. 2026 Aug 6;18(15):2570. doi: 10.3390/nu18152570 (PMC13467854; doi:10.3390/nu18152570)
Supplement: Supplementary file 1 [file nutrients-18-02570-s001.zip › nutrients-4393129-supplementary.pdf]

Supplemental Table S1. Food Categories by BNS codes [1].

Food Categories	BNS Codes	Descriptions
	PASTA, RICE, CEREAL GRAINS AND FLOUR - 01	
1	01A	pasta
1	01B	rice
1	01C	cereal grains and flours
1	130A	those in the ingredient column - we think this is an error in the database, but this is spaghetti (1 food entry)
	WHITE BREADS - 02	
2	02A	white bread
	WHOLEMEAL BREADS - 03	
3	03A	whole wheat bread
3	03B	other whole grains
	OTHER BREADS - 04	
4	04A	rolls, bagels, pita bread, croutons, dumplings, matzo, tortilla
4	04B	crackers and crispbreads
4	04C	muffin and english muffin
4	04D	pancakes and waffles
4	04E	croissants, piecrusts & phyllo dough
4	04F	dry mixes (cakes, muffins, pancakes)
4	219B	those in the ingredient column - we think this is an error in the database, but this is submarine (1 entry)
	WHOLEGRAIN AND HIGH FIBRE BREAKFAST CEREALS - 05	
5	05A	whole grain, oats and high fibre breakfast cereals
	OTHER BREAKFAST CEREALS - 06	
6	06A	breakfast cereals
	COOKIES, BISCUITS AND GRANOLA BARS - 07 (+SUG150B COOKIE)	
7	07A	cookies, commercial
7	07B	biscuits, commercial
7	07C	granola bar
7	150B	cookie
	CAKES, PIES, DANISHES AND OTHER PASTRIES - 08 (+SUG150 RECIPES EXCEPT COOKIE IN ABOVE)	
8	08A	pies, commercial
8	08B	cakes, commercial (frozen cake)
8	08C	danishes, doughnuts and other pastries, commercial
8	150A	cakes, cheesecakes, shortcakes and brownies
8	150C	danishes, turnovers & pastries
8	150D	donuts
8	150E	muffins
8	150F	pies (incl pie shell)
8	150G	squares & bars
8	150H	sweet rolls and breads

**8**	150I	filled crepes, blintzes, cobblers
	FROZEN DAIRY PRODUCTS - 09	
**9**	09A	ice cream
**9**	09B	ice milk
**9**	09C	frozen yoghurt
	MILKS - 10	
**10**	10A	milk, whole
**10**	10B	milk, 2%
**10**	10C	milk, 1%
**10**	10D	milk, skim
**10**	10E	milk, evaporated, whole
**10**	10F	milk, evaporated, 2%
**10**	10G	milk, evaporated, skim
**10**	10H	milk, condensed
**10**	10I	other types of milk (whey, buttermilk)
**10**	10J	plant-based beverage (soy, almond, coconut)
**10**	10K	goat and sheep milk
	CREAMS - 13	
**13**	13A	whipping cream
**13**	13B	table cream
**13**	13C	half & half cream
**13**	13D	sour cream
	CHEESES - 14	
**14**	14A	cottage cheese
**14**	14B	cheese, less than 10% B.F.
**14**	14C	cheese, 10% B.F. to 25% B.F.
**14**	14D	cheese, more than 25% B.F.
	YOGURTS (NATURAL AND WITH FRUIT) - 15	
**15**	15A	yoghurts, less than 2% B.F.
**15**	15B	yoghurts, more than 2.1% B.F.
	EGGS - 16	
**16**	16A	egg
**16**	16B	egg substitutes
	BUTTERS - 17	
**17**	17A	butter
	MARGARINES + OTHER FATS & SPREADS - 18, 20, 21 = 18	
**18**	18A	regular tub margarine
**18**	18B	calorie-reduced tub margarine
**18**	20A	block margarine
**18**	21A	vegetable oils
**18**	21B	animal fats
**18**	21C	shortening
**18**	227A	those in the ingredient column - we think this is an error in the database, but these are all fats and oils
	RED MEATS - 22, 23, 24, 25, 28, 29, 30, 31, 32 = "22"	

22	22A	beef, lean only
22	22B	beef, lean + fat
22	22C	beef, ground
22	23A	veal, lean only
22	23B	veal, lean + fat (incl ground veal)
22	24A	lamb, lean only
22	24B	lamb, lean + fat (incl ground lamb)
22	25A	pork, fresh, lean only
22	25B	pork, fresh, lean + fat
22	25C	bacon
22	25D	ham, cured, lean only
22	25E	ham, cured, lean + fat
22	28A	liver
22	28B	liver pate
22	29A	offal
22	31A	game meat
	SAUSAGE + LUNCHEON MEATS (SOME SUGAR ADDED)	
23	30A	sausage
23	32A	luncheon meat
	POULTRY - 27	
27	27A	chicken, meat only
27	27B	chicken, meat + skin
27	27C	turkey, meat only
27	27D	turkey, meat + skin (incl ground turkey)
27	27E	other birds (duck, pheasant, pigeon)
27	27F	birds, skin only
	NUTS, SEEDS AND PEANUT BUTTER - 33	
33	33A	nuts
33	33B	seeds
33	33C	peanut butter and other nut spreads
	FISH + SHELLFISHES - 34, 35 - "34"	
34	34A	fish, less than 6% total fat
34	34B	fish, superior or equal to 6% total fat
34	35A	shellfish
	VEGETABLES - 36	
36	36A	beans
36	36B	broccoli
36	36C	cabbage and kale
36	36D	cauliflower
36	36E	carrots
36	36F	celery
36	36G	corn
36	36H	lettuces & leafy greens (spinach, mustard greens, etc.)
36	36I	mushrooms
36	36J	onion, green onions, leeks, garlic

**36**	36K	peans and snow peas
**36**	36L	peppers, red & green
**36**	36M	squashes
**36**	36N	tomatoes
**36**	36O	juices, tomato & vegetable
**36**	36P	other veg (cucumber, immature beans, brussel sprouts, beets, turnips)
	VEG SOUPS - 501	
**501**	50A	soups with vegetables
**501**	50B	soups without vegetables
	LEGUMES - 37	
**37**	37A	legume
**37**	37B	foods made with vegetable proteins (tofu)
	POTATOES, FRIED - 38	
**38**	38A	potato chips
**38**	38B	fried or roasted potatoes
	POTATOES, RAW AND COOKED (EXCLUDING FRIED) - 39	
**39**	39A	potato
	FRUIT - 40	
**40**	40A	citrus fruits
**40**	40B	apple
**40**	40C	banana
**40**	40D	cherries
**40**	40E	grapes and raisins
**40**	40F	melons
**40**	40G	peaches, nectarines
**40**	40H	pears
**40**	40I	pineapple
**40**	40J	plums and prunes
**40**	40K	strawberries
**40**	40L	other fruits (blueberries, dates, kiwis, fruit salads, etc)
	SUGARS, SYRUPS AND PRESERVES - 41	
**41**	41A	sugars (white and brown)
**41**	41B	jams, jellies and marmalade
**41**	41C	other sugars (syrups, molasses, honey, etc)
**41**	41D	sugar substitutes
	SAVORY SNACKS - 42	
**42**	42A	popcorn, plain & pretzels
**42**	42B	salty and high fat snacks (incl tortilla chips)
	CONFECTIONARY - 43 + 44 - "43"	
**43**	43A	candies, gums, etc
**43**	43B	ice pop, sherbert
**43**	43C	gelatin, dessert toppings and pudding mixes, commercial
**43**	44A	chocolate bar
	FRUIT JUICE - 45	

**45**	45A	fruit juice
	ALCOHOLIC BEVERAGES - 47, 48, 49 - "47"	
**47**	47A	spirits
**47**	47B	liqueurs
**47**	48A	wine
**47**	49A	beer
**47**	49B	ciders and coolers
	CONDIMENTS + GRAVIES - "50"	
**50**	50C	gravies
**50**	50D	sauces (white, bearnaise, soya, tartar, ketchup, etc)
**50**	50E	salad dressings (with or without oil)
**50**	50F	seasonings (salt, vinegar, etc)
**50**	53A	spices
**50**	53B	others (baking soda, baking power, yeast, etc)
	TEA + COFFEE - "51"	
**51**	51A	tea (incl iced tea)
**51**	51B	coffee
**51**	51C	water
**51**	231B	tea (recipe sub-group)
**51**	231C	coffee (recipe sub-group)
	BABYFOOD PRODUCTS - 52	
**52**	52A	babyfood product
**52**	SUG52B	infant formula
	SUPPLEMENTED BARS, SHAKES AND MEAL REPLACEMENTS - 54	
**54**	54A	energy bar
**54**	54B	protein bar and shake
**54**	54C	meal replacements
	SOFT DRINKS - REGULAR - 60	
**60**	46A	soft drinks - regular
	SOFT DRINKS - DIET - 61	
**61**	46B	soft drinks - diet
	FRUIT DRINKS - 62	
**62**	46C	fruit drinks
	OTHER BEVERAGES - 46D-G - 63	
**63**	46D	other beverages (malted milk, chocolate beverage)
**63**	46E	energy drink
**63**	46F	vitamin water
**63**	46G	sports drink
**63**	231D	milk-based beverage (milk shakes, malted milk, hot cocoa, instant breakfast, etc)

[1]BNS codes beyond 99 are recipes, and are only included if they are apparent sources of added sugars.

Supplementary Table S2. The 10-step algorithm to estimate added sugars, adapted from Louie et al. [14].

Step 1. Does the food contain 0 g total sugar?
├─── Yes → Assign 0 g added sugar.
└─── No → Proceed to Step 2.

Step 2. Does the food belong to one of the no added sugar food groups?
── Yes → Assign 0 g added sugar.
└─── No → Proceed to Step 3.

Step 3. Does the food belong to one of the 100% added sugar food groups?

── Yes → Assign added sugar = 100% of total sugar.
└─── No → Proceed to Step 4.

Step 4. Are added sugar content of all ingredients in the standard recipe known?
├─── Yes → Calculate added sugar based on proportioning method

└─── No → Proceed to Step 5.

Step 5. Is there a comparable unsweetened variety of the food?
├─── Yes → Calculate added sugar based on unsweetened variety method
└─── No → Proceed to Step 6.

Step 6. Are analytical data of individual sugar types available?
├─── Yes → Estimate based on analytical data.
└─── No → Proceed to subjective estimation.

Step 7. Are there any similar products with known added sugar content (from overseas database or from steps 1 – 6)?
├─── Yes → Borrow value from similar foods.
└─── No → Proceed to Step 8.

Step 8. Could the added sugar content of the food be subjectively estimated based on available information?
├─── Yes → Subjectively estimate the added sugar contents.
└─── No → Proceed to Step 9.

Step 9. Are added sugar content of all ingredients in the standard recipe known after steps 5 - 8?
├─── Yes → Calculate added sugar based on proportioning method.
└─── No → Proceed to Step 10.

Step 10. 50% of the total sugars as added sugars

Supplementary Table S3. HEFI component score algorithm (adapted from Brassard et al. 2022) [9].

Component	Measurement (ratio)	Maximum Points	Standard for minimum score	Standard for maximum score
Vegetable and fruits	Vegetable and fruits (RA^) /total foods (RA)	20	No vegetable and no fruits	Ratio ≥ 0.5
Whole-grain foods	Whole-grain foods (RA)/total foods (RA)	5	No whole grain foods	Ratio ≥ 0.25
Grain foods ratio	Whole-grain foods (RA)/total grain foods (RA)	5	No whole grain foods	Ratio = 1.0
Protein foods	Protein foods (RA)/total foods (RA)	5	No protein foods	Ratio ≥ 0.25
Plant-based protein foods	Plant-based protein foods (RA)/total protein foods (RA)	5	No plant-based protein foods	Ratio ≥ 0.5
Beverages	Plain water*/total beverages	10	No plain water	Ratio = 1.0
Fatty acid ratio	Unsaturated#/saturated fatty acids	5	Ratio ≤ 1.1	Ratio ≥ 2.6
Saturated fat	Saturated fatty acid (%energy intake)	5	≥ 15%	< 10%
Free sugars	Free sugars (%energy intake)	10	≥ 20%	< 10%
Sodium	Total sodium (mg/kcal)	10	>2.0	<0.9

^Reference Amount

*Including carbonated and unsweetened beverages

#Monounsaturated+polyunsaturated fatty acids

Supplementary Table S4. Energy adjustment model selection

Model type	Sugar variable	R^2	AIC	BIC	P-value
Nutrient residual model					
GLM	residual	0.2528	110229	90552	All significant

Energy partition model					
GLM	Energy from sugar and residual energy	0.2528	110229	90552	All significant
Nutrient density model					
GLM	Percentage of energy from sugar	0.2954	107743	88317	All significant

*In the nutrient density model, added sugars are expressed as a proportion of energy (% of energy intake from added sugars). In the energy partition model, total calories from all sources, excluding added sugars, are included in the model as a covariate. In the residual model, residuals are obtained by regressing added sugars on total energy intake; these residual values are then centered on the predicted added sugars intake corresponding to the mean energy intake of the study population, and the model is fitted using these centered residuals along with total energy intake.

Supplementary Table S5. The regression models tested for the relationship between added sugars and HEFI scores

Model A	Linear with continuous independent variable	The added sugar intake variable as the main independent variable in the analysis to assess the presence of a linear relationship with HEFI score.
Model B.1	Linear with categorical independent variable	The added sugar intake as %EI variable was separated into quintiles as the main independent variable in the analysis to assess the presence of a linear relationship with HEFI score.
Model B.2	Linear with categorical independent variable	The added sugar intake as %EI variable was separated into different groups using the thresholds of <5% EI, 5-10% EI, 10-15% EI, 15-20% EI, and over 20% EI.
Model C	Curve Linear	The quadratic term of the added sugar intake variable was added to Model A to further explore the possibility of a non-linear, curve linear association with risk of HEFI score.

Supplementary Table S6. Regression model selection results.

Model type	Sugar variable	R^2	AIC (AICR for robust regression)	BIC (BICR for robust regression)	P value
GLM	AS	0.309	108700	89023	All significant

	categorical (Quintile)				
GLM	AS categorical (Sugar group)	0.3168	107148	87722	All significant
GLM	AS continuous	0.2935	109129	89453	All significant
GLM with HEFI residue	AS continuous	0.1305	106205	86779	All significant except education level2
GLM	Quadratic term and continuous	0.3126	108593	88916	All significant
Robust regression	AS continuous	0.2650	16494	16586	All significant
	AS categorical	0.2765	16445	16562	All significant
	Quadric	0.2799	16608	16708	All significant
	Log term for HEFI and sugar	0.228	17803	17894	All significant

Supplementary Table S7. Key demographic characteristics of the study population.

Sex (%)	Male	48.1%
	Female	51.9%
Age Group (%)	2-8 y	10.9%
	9-13 y	9.9%
	14-18 y	9.8%
	19-30 y	9.1%
	31-50 y	21.1%
	50+ y	39.1%
Education (%)	Less than high school diploma or equivalent	40.0%
	High school diploma or a high school equivalency certificate	23.1%
	Certificate/diploma - trade/college/non-university/university below Bachelor	19.8%
	Bachelor's degree or university certificate/diploma/degree above Bachelor level	16.6%
	missing	0.5%
Misreporting status (%)	Plausible	60.0%
	Under-reporting	29.5%
	Over-reporting	10.5%
Smoking status (%)	Never smoke	85.9%
	Occasionally smoke	10.7%
	Daily smoke	3.4%

Supplementary Table S8. Distribution of the study population by added sugars intake categories.

		0-5% EI	5-10% EI	10-15% EI	15-20% EI	20+% EI	P value (Chi^2)
Sex (Count)	Male	32.5%	28.3%	18.6%	10.4%	10.2%	<0.0001
	Female	31.7%	30.3%	18.7%	9.5%	9.7%	

(%)							
Education (Count) (%)	Less than high school diploma or equivalent	25.7%	30.0%	21.2%	11.5%	11.7%	<0.0001
	High school diploma or a high school equivalency certificate	33.5%	27.4%	18.4%	9.7%	11.0%	
	Certificate/diploma - trade/college/non-university/university below Bachelor	36.2%	29.3%	16.3%	9.2%	9.1%	
	Bachelor's degree or university certificate/diploma/ degree above Bachelor level	39.9%	30.4%	16.0%	7.8%	5.9%	
	missing	44.3%	26.4%	22.6%	2.8%	3.8%	
Misreporting status (Count) (%)	Plausible	29.8%	30.7%	20.0%	10.3%	9.1%	<0.0001
	Under-reporting	38.7%	25.9%	15.8%	8.7%	10.9%	
	Over-reporting	26.7%	31.4%	18.9%	11.2%	11.8%	
Smoking status (Count) (%)	Never smoke	32.4%	30.0%	19.2%	9.7%	8.7%	<0.0001
	Occasionally smoke	34.2%	30.3%	14.1%	10.8%	10.6%	
	Daily smoke	29.4%	23.8%	15.7%	11.9%	19.3%	

*With bootstrap (BRR) and weight applied.

Supplementary Table S9. Generalized Linear Model's coefficient, %95 CI, and P-value between HEFI and key demographic groups.

**Sex**	**Male (reference)**	**0**	**0**	
	Female	2.12	1.61,2.63	<0.0001
**Education level**	Less than high school diploma or equivalent (reference)	0	0	.
	High school diploma or a high school equivalency certificate	1.08	-0.01,2.18	0.0531
	Certificate/diploma - trade/college/non-university/university below Bachelor	1.12	0.21,2.03	0.0164
	Bachelor's degree or university certificate/diploma/degree above Bachelor level	3.22	2.45,4.00	<0.0001
**Smoking status**	Never smoke (reference)	0	0	.
	Occasionally smoking	-2.93	-6.41,0.55	0.0987
	Daily smoke	-3.38	-4.39,-2.36	<0.0001
**Misreporting**	Plausible (reference)	0	0	.
	Over-reporting	-2.16	-3.20,-1.12	<0.0001
	Under-reporting	0.73	0.11,1.35	0.0212

Supplementary Figures S1. Sensitivity analysis results with underweight population included.

Estimated Regression Coefficients						
Parameter	Estimate	Standard Error	t Value	Pr > |t|	95% Confidence Interval	
Intercept	44.333025	0.47412682	93.50	<.0001	43.401499	45.264552
sugargrp 10-15% EI	-7.411798	0.35698919	-20.76	<.0001	-8.113181	-6.710414
sugargrp 15-20% EI	-13.236694	0.95098952	-13.92	<.0001	-15.105122	-11.368266
sugargrp 20+% EI	-17.611276	0.78890998	-22.32	<.0001	-19.161263	-16.061289
sugargrp 5-10% EI	-3.271267	0.33880467	-9.66	<.0001	-3.936924	-2.605611
sugargrp 0-5% EI	0.000000	0.00000000	.	.	0.000000	0.000000
age	0.054497	0.01002791	5.43	<.0001	0.034795	0.074199
sex 2	2.098409	0.25689829	8.17	<.0001	1.593676	2.603142
sex 1	0.000000	0.00000000	.	.	0.000000	0.000000
education 2	1.046514	0.55657785	1.88	0.0607	-0.047006	2.140033
education 3	1.093088	0.45721901	2.39	0.0172	0.194780	1.991395
education 4	3.181310	0.38754446	8.21	<.0001	2.419893	3.942726
education 1	0.000000	0.00000000	.	.	0.000000	0.000000
smoking 1	-2.792144	1.68252419	-1.66	0.0976	-6.097832	0.513545
smoking 2	-3.452051	0.50283151	-6.87	<.0001	-4.439974	-2.464128
smoking 0	0.000000	0.00000000	.	.	0.000000	0.000000
Misreporting Over-reporting	-2.157548	0.52918426	-4.08	<.0001	-3.197247	-1.117849
Misreporting Under-reporting	0.729692	0.31450353	2.32	0.0207	0.111781	1.347603
Misreporting Unknown	0.202495	0.97234545	0.21	0.8351	-1.707891	2.112881
Misreporting Plausible	0.000000	0.00000000	.	.	0.000000	0.000000

Supplementary Figure S2. Non-linear relationship between HEFI and variables (% from added sugars and age).

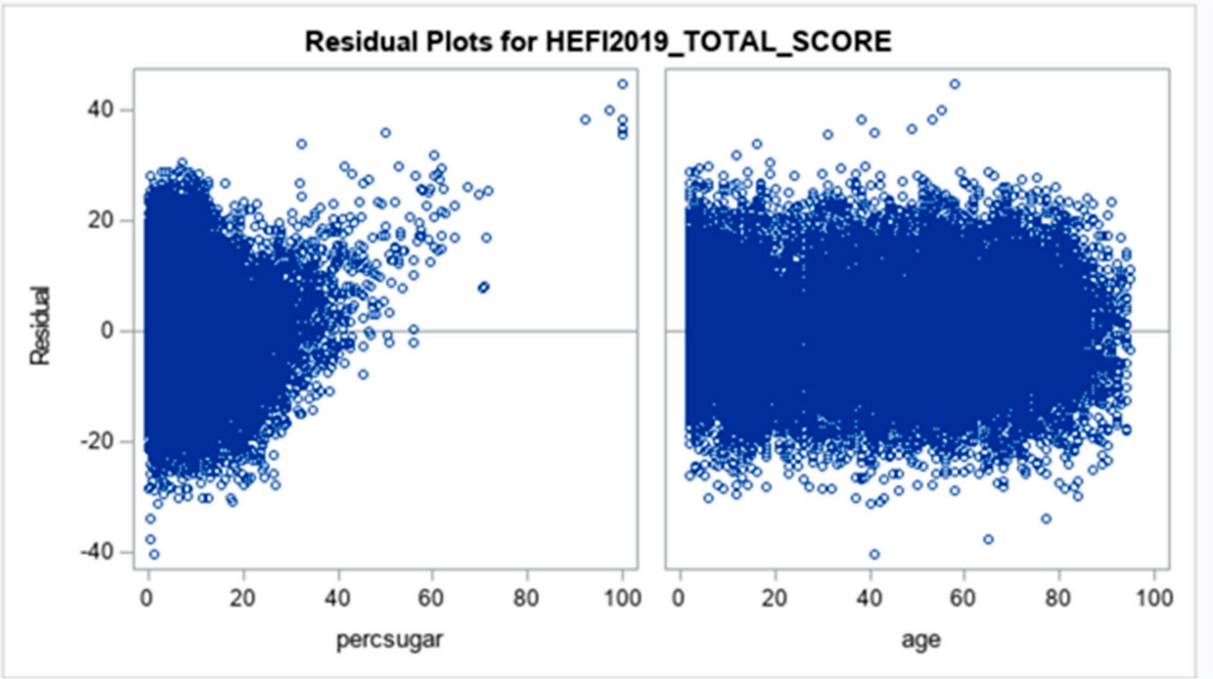

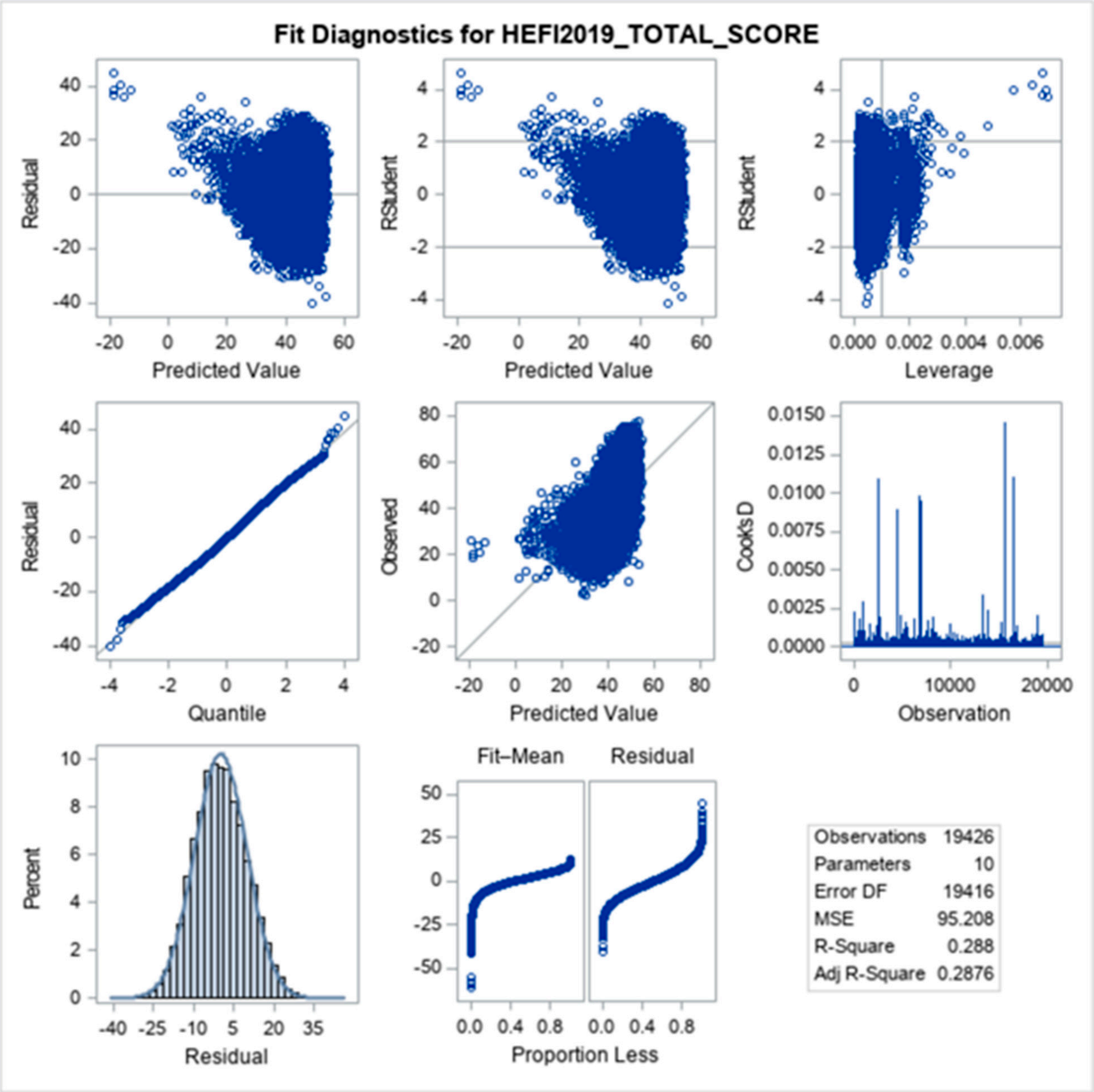
Fit Diagnostics for HEFI2019_TOTAL_SCORE
Residual
Predicted Value
RStudent
Predicted Value
RStudent
Leverage
Residual
Quantile
Observed
Predicted Value
Cook's D
Observation
Percent
Residual
Fit–Mean
Residual
Proportion Less
Observations 19426
Parameters 10
Error DF 19416
MSE 95.208
R-Square 0.288
Adj R-Square 0.2876

Supplementary Figure S3. Sensitivity analysis with or without participants with added sugars greater than 40% EI.

1). With outlier, not weighted

Parameter Estimates					
Parameter	DF	Estimate	Standard Error	t Value	Pr > |t|
Intercept	1	49.256386	0.213103	231.14	<.0001
sugargrp	1	-4.300601	0.054002	-79.64	<.0001
age	1	0.045696	0.003140	14.55	<.0001
sex 2	1	2.029576	0.137869	14.72	<.0001
sex 1	0	0	.	.	.
education 2	1	0.543545	0.204065	2.66	0.0077
education 3	1	1.025881	0.196257	5.23	<.0001
education 4	1	3.423398	0.214362	15.97	<.0001
education 1	0	0	.	.	.
smoking 1	1	-2.166725	0.383455	-5.65	<.0001
smoking 2	1	-3.948529	0.228521	-17.28	<.0001
smoking 0	0	0	.	.	.
Misreporting Over-reporting	1	-1.666656	0.230007	-7.25	<.0001
Misreporting Under-reporting	1	0.672931	0.155452	4.33	<.0001
Misreporting Plausible	0	0	.	.	.

2). Without outlier, not weighted

Parameter Estimates					
Parameter	DF	Estimate	Standard Error	t Value	Pr > |t|
Intercept	1	49.195010	0.214496	229.35	<.0001
sugargrp	1	-4.277431	0.054907	-77.90	<.0001
age	1	0.045493	0.003153	14.43	<.0001
sex 2	1	2.036111	0.138550	14.70	<.0001
sex 1	0	0	.	.	.
education 2	1	0.549604	0.205387	2.68	0.0075
education 3	1	1.048182	0.197202	5.32	<.0001
education 4	1	3.449056	0.215277	16.02	<.0001
education 1	0	0	.	.	.
smoking 1	1	-2.158032	0.385285	-5.60	<.0001
smoking 2	1	-3.945343	0.231425	-17.05	<.0001
smoking 0	0	0	.	.	.
Misreporting Over-reporting	1	-1.689069	0.230860	-7.32	<.0001
Misreporting Under-reporting	1	0.727145	0.156477	4.65	<.0001
Misreporting Plausible	0	0	.	.	.

3). Without outlier, weighted

Parameter Estimates					
Parameter	DF	Estimate	Standard Error	t Value	Pr > |t|
Intercept	1	49.195010	0.214496	229.35	<.0001
sugargrp	1	-4.277431	0.054907	-77.90	<.0001
age	1	0.045493	0.003153	14.43	<.0001
sex 2	1	2.036111	0.138550	14.70	<.0001
sex 1	0	0	.	.	.
education 2	1	0.549604	0.205387	2.68	0.0075
education 3	1	1.048182	0.197202	5.32	<.0001
education 4	1	3.449056	0.215277	16.02	<.0001
education 1	0	0	.	.	.
smoking 1	1	-2.158032	0.385285	-5.60	<.0001
smoking 2	1	-3.945343	0.231425	-17.05	<.0001
smoking 0	0	0	.	.	.
Misreporting Over-reporting	1	-1.689069	0.230860	-7.32	<.0001
Misreporting Under-reporting	1	0.727145	0.156477	4.65	<.0001
Misreporting Plausible	0	0	.	.	.

Supplementary Figure S4. Weighted distribution of HEFI scores (with medians and interquartile ranges) across AS intake categories and age groups.

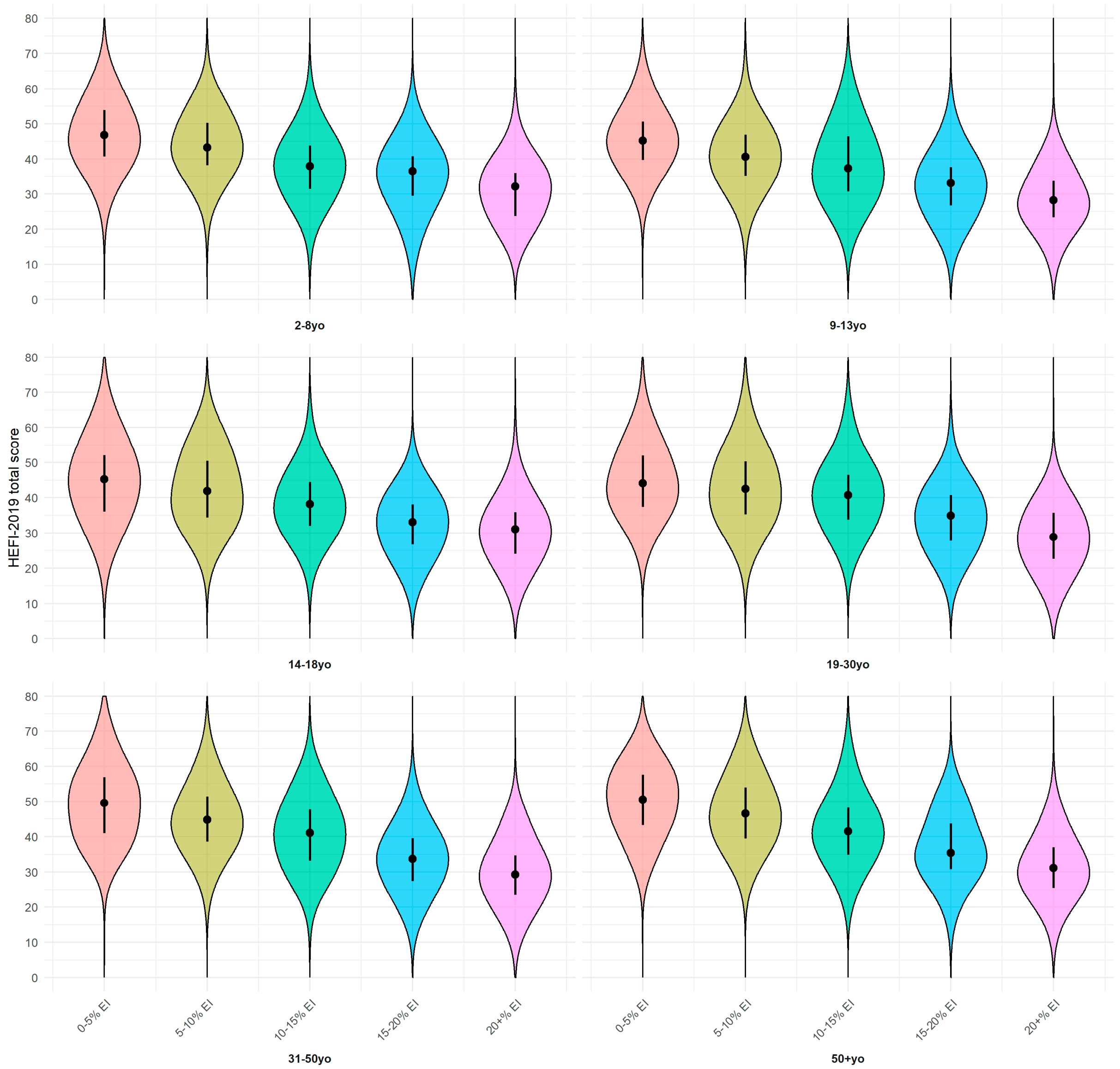

Age group	Added Sugars Category	Median_IQR
2-8yo	0-5% EI	46.8(40.7,53.9)
	5-10% EI	43.3(38.2,50.3)
	10-15% EI	38.0(31.4,43.8)
	15-20% EI	36.5(29.5,40.8)
	20+% EI	32.1(23.7,36.0)
9-13yo	0-5% EI	45.2(39.7,50.6)

	5-10% EI	40.6(35.2,46.9)
	10-15% EI	37.3(30.7,46.4)
	15-20% EI	33.1(26.7,37.7)
	20+% EI	28.2(23.4,33.7)
14-18yo	0-5% EI	45.3(36.1,52.0)
	5-10% EI	41.9(34.4,50.5)
	10-15% EI	38.1(32.0,44.5)
	15-20% EI	33.0(26.7,38.1)
	20+% EI	31.0(24.0,35.9)
19-30yo	0-5% EI	44.1(37.3,52.0)
	5-10% EI	42.5(35.3,50.2)
	10-15% EI	40.7(33.7,46.5)
	15-20% EI	34.9(27.8,40.7)
	20+% EI	28.8(22.6,35.7)
31-50yo	0-5% EI	49.5(41.0,56.9)
	5-10% EI	44.7(38.6,51.4)
	10-15% EI	41.0(33.2,47.7)
	15-20% EI	33.8(27.5,39.5)
	20+% EI	29.3(23.5,34.7)
50+yo	0-5% EI	50.5(43.3,57.6)
	5-10% EI	46.5(39.4,54.0)
	10-15% EI	41.5(34.9,48.2)
	15-20% EI	35.4(30.8,43.7)
	20+% EI	31.2(25.5,37.0)
